# Fetal microchimerism in mouse caerulein-induced pancreatitis model 

**DOI:** 10.22038/IJBMS.2018.26976.6595

**Published:** 2018-09

**Authors:** Zahra Vojdani, Jafar Bagheri, Tahereh Talaei-Khozani, Negar Azarpira, Mahin Salmannjad, Ali Farrokhi

**Affiliations:** 1 Laboratory for Stem Cell Research, Anatomy Department, Medical School, Shiraz University of Medical Sciences, Shiraz, Iran; 2 Transplantation Research Center, Shiraz University of Medical Sciences, Shiraz, Iran; 3 Royan Institute for Stem Cell Biology and Technology, ACECR Department of Stem Cell and Developmental Biology, Cell Science Research Center, Tehran, Iran

**Keywords:** Acute pancreatitis, Caerulein, Chimerism, Enhanced green fluorescent- protein, Mice, Pregnancy

## Abstract

**Objective(s):**

Fetal microchimerism is the persistence of allogeneic cell population that transfer from the fetus to the mother. The aim of this study was to evaluate the presence of fetal microchimerism in the pancreas of the mouse with acute pancreatitis (AP).

**Materials and Methods:**

In this experimental study, female wild-type mice were mated with male EGFP+. AP model was obtained by injection of caerulein two days after delivery. Sixty mice were divided into 3 groups: the virgin pancreatitis-induced animals, pregnant pancreatitis-induced animals mated with transgenic EGFP mice, and pregnant sham animals. To prove pancreatitis induction, the blood amylase and lipase were assessed; and pancreas was removed from a subpopulation of each group for histopathological examinations after 6 hr. The remaining mice were kept for 3 weeks and histopathological exanimation, immunohistochemistry, and PCR were performed.

**Results:**

EGFP+ cells were found in acini and around the blood vessels in the pancreas of pregnant pancreatitis-induced animals. They differentiated to acinar, adipocyte-like, and mesenchymal-like cells. PCR showed that 20% of the pregnant pancreatitis-induced animals were EGFP+. The histopathological study showed improvement in pancreatitis scores in the mice with history of pregnancy.

**Conclusion:**

It seems that pregnancy has a beneficial impact on caerulein-induced pancreatitis and improves the pancreatitis score in mouse.

## Introduction

Microchimerism is defined as the long-term persistence of a population of allogeneic cells (or DNA) (1) within an individual (2). Microchimerism happens as a result of the reciprocal trafficking of the cells between the fetus and mother during pregnancy, transfusion between twins, organ transplantation, and blood transfusion (3, 4). Microchimerism may have a physiological role in tissue repair, is associated with autoimmune diseases (5), or is involved in some cancers (3).

Most of the microchimerism was observed during pregnancy (4). Microchimerism can be divided into two types: the cells that are transferred from the fetus to mother, called fetal microchimerism cells (FMC), and the cells that are transferred from the mother to fetus, called maternal microchimerism cells. Both of these cells can remain in the blood and tissues for decades or even the whole life (6).

In humans, FMC is the most common form of microchimerism. Although FMC has been reported to happen even before the formation of the placenta (7), it has been detected to be a result of passing the fetal cells through the placenta (8) at four to six (9) and seven weeks of gestation. FMC has been derived from hematopoietic stem cells (10), hematopoietic cell lineages including B and T lymphocytes, monocytes, natural killer cells, CD34^+ ^(11), CD38^+ ^(12), macrophages and granulocytes (1), red blood cells, or mesenchymal stem cells (13). They also have been derived from the trophoblast cells, primitive endoderm, and epiblast. The cells enter the maternal circulation and transport to the bone marrow, proliferate in the body, remain in the mother’s body for decades (14, 15), and differentiate into several cell types (16). In maternal tissues, these cells have been traced as neurons and neuroglia (11), hepatocytes, kidney’s tubular epithelium (17), cardiomyocytes (18), intestinal epithelium, endothelial cells (10), and thyrocytes (11) as well as in organs such as the lung (13), skin, spleen, adrenal gland (18), appendix (19), and spinal cord (20).

Various hypotheses suggest the roles and functions of FMC in the mother’s body. FMC may trigger graft versus host reaction that leads to autoimmune diseases (11). FMC has been suggested to be involved in autoimmune diseases such as scleroderma, primary biliary cirrhosis, systemic lupus erythematosus, myositis, Sjogren’s syndrome (21), rheumatoid arthritis (22, 23), type I diabetes (24), Hashimoto’s thyroiditis, Grave’s disease (9), and multiple sclerosis (6). Long-term presence of FMC in the mother’s body increases the capability of the maternal immune system to respond to FMC and this may lead to various immunologic diseases in the mothers (12). 

Other evidence suggests that exchanging cells in both types of microchimerism have a role in triggering non-autoimmune diseases, such as cancer in the thyroid, cervix, lung (1), breast, colon, uterus, ovary (6), and also in melanoma (25) and hepatitis C (26).

It has been also hypothesized that FMC is distributed in maternal tissues following injury, acquires stemness activities, and participates in tissue repair. After delivery, these cells are suggested to be placed in the maternal stem cells niche (12). These cells differentiate and start regenerative responses in damaged maternal tissues (10, 27). FMC has been shown to differentiate into hepatocytes and renal tubular cells in the maternal liver and kidney, and is also involved in tissue repair (17). The presence of fetal cells in the maternal heart was also reported after cardiomyocyte inflammation. FMCs were shown to migrate to the myocardium and differentiate into cardiomyocytes (18). FMC migration to the lung and bone marrow has been detected also in mouse (13). Interestingly, the fetal cells have been shown to pass through the blood-brain barrier and, acquire different morphology in response to brain injury (31), depending on the position. Fetal cells also differentiate into neurons, astrocytes or oligodendrocytes in the spinal cord (20).

Acute and chronic pancreatitis are common gastrointestinal disorders that are very important in terms of reducing the quality of life for patients. Acute pancreatitis causes inappropriate activation of pancreatic zymogen (including trypsinogen) and increas lysosomal enzymes (including cathepsin) in the pancreas, which leads to cell death and necrosis of the pancreatic cells. It also causes extensive inflammatory response and release of inflammatory mediators such as TNF-α, IL-1, IL-6, and neutrophil infiltration in the pancreas (29, 30). In acute pancreatitis, mitochondria are disabled, increasing the number and size of autophagy vesicles.

The development of pancreatitis animal models has increased our understanding of pathogenesis and pathophysiology. They include models consisting of secretagogue hyperstimulation (caerulein injection), ethanol feeding, bile salt duct infusion, choline-deficient ethionine-supplemented, basic amino acid, specific cytokines injection, and infection due to coxsackie virus group B (31).

The aim of this study was to investigate the presence of FMCs in the pancreatic tissues of mice afflicted with acute pancreatitis. This study for the first time shows the positive effects of pregnancy in acute pancreatitis. 

## Materials and Methods

***Animals***

This work was an interventional experimental study. All animals were handled according to the guidelines approved by ethics committee of Shiraz University of Medical Sciences. Transgenic EGFP adult male mice (NMRI strain), weighing 25–30 g (Royan Institute) and wildtype female virgin mice weighing 25–30 g (purchased from the animal lab of Shiraz University of Medical Sciences) were selected randomly. Animals were kept in standard conditions (12 hr light/ dark period, temperature of 18–22 ^°^C) with free access to food and water.

**Table 1:** EGFP Primer sequence

Gene	Primer	Primer sequence	Length
**EGFP**	Forward	5'CGTAAACGGCCACAAGTTC 3'	19bp
	Reverse	5'TTGAAGAAGTCGTGCTGC 3'	18bp

**Figure1:**
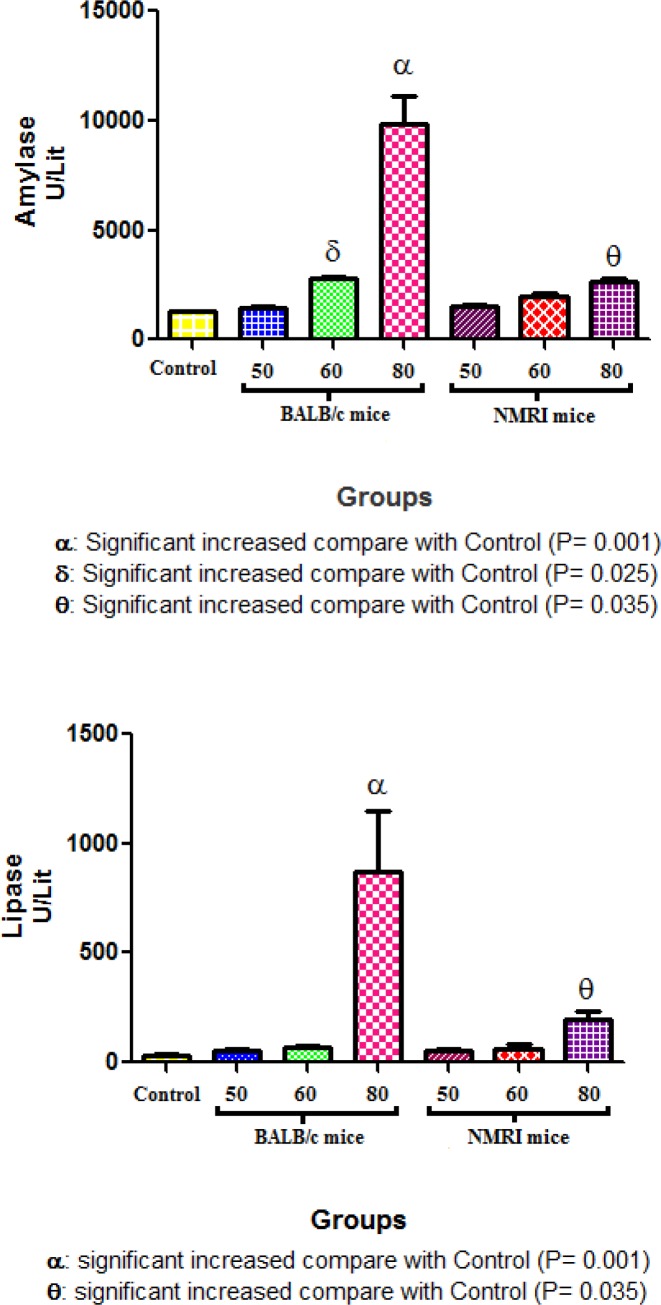
The pilot study showed that the activity of amylase and lipase (U/ml) 6 hr after the last injections were significantly higher in the mice receiving 80 µg/kg caerulein. The serological tests showed that injection of 80 µg/kg caerulein-induced higher amylase and lipase activities in balb/C than in NMRI mice

**Figure 2:**
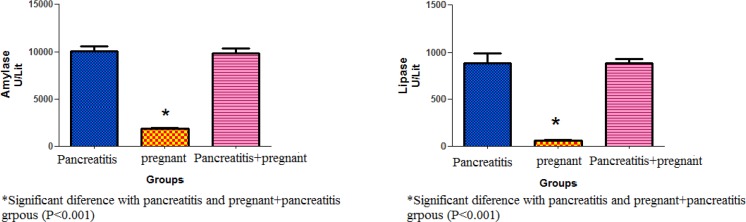
The activity of amylase and lipase (U/mL) 6 hr after the last caerulein injections in different groups. The amylase and lipase activity increased significantly in the sera of both caerulein-injected animals, which confirmed pancreatitis induction

**Figure 3:**
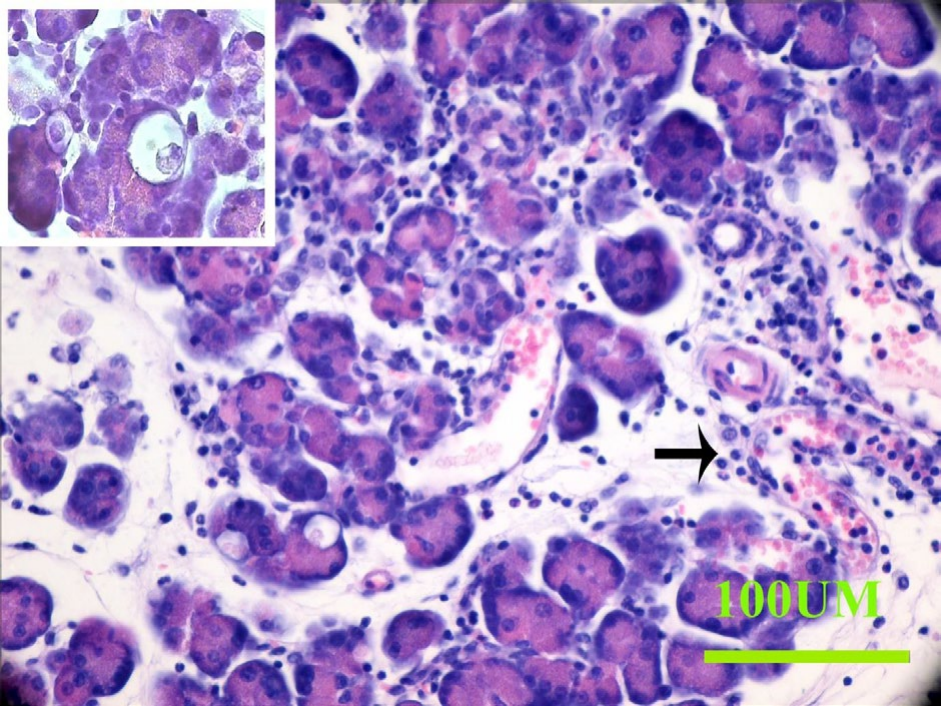
The micrographs show the pathological changes 6 hr after the induction of pancreatitis. Leukocyte infiltration (arrow) and acinar vacuolization (left above) are shown

**Figure 4:**
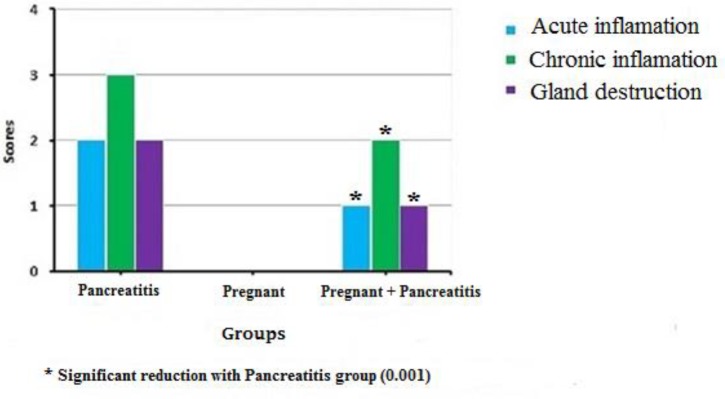
The comparison of the median value of scoring of chronic, acute, and gland destruction in different groups 3 weeks after pancreatitis induction. The pregnancy caused a significant reduction in the median value of the scores in the pancreatitis-induction animals

**Figure 5:**
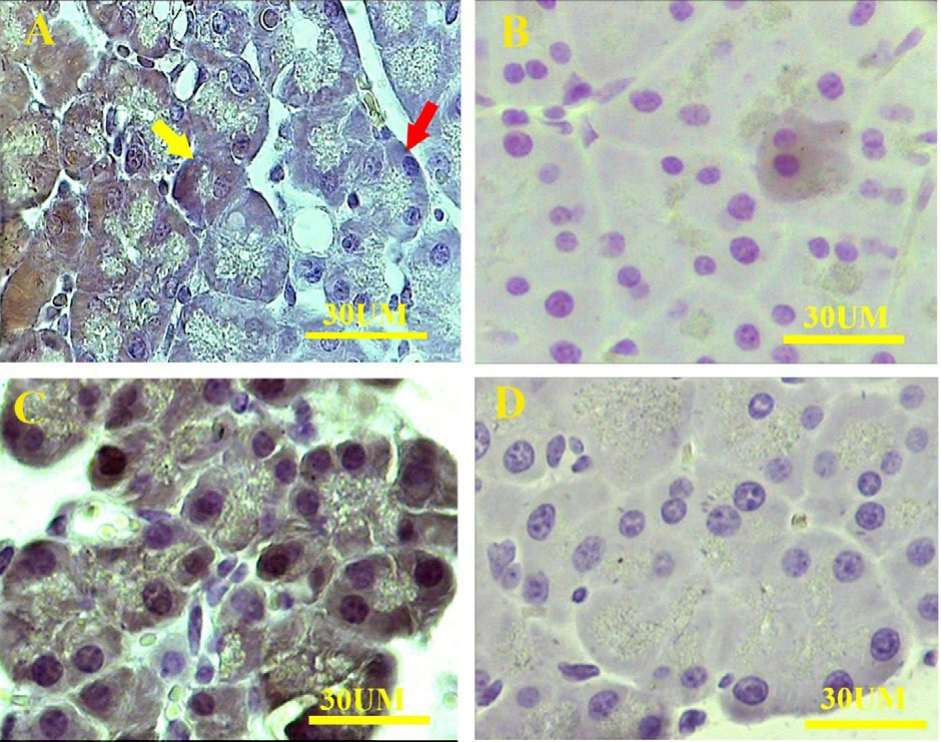
Immunostaining of the pancreas with anti-GFP antibody. A; EGFP-positive acinar cells of pregnant pancreatitis-induced (yellow arrow) along with EGFP-negative acini (red arrow) are present in some cases. B; a few EGFP-positive acinar cells are present in pregnant-sham animals. The number of the EGFP-positive cells is more in pregnant pancreatitis-induced animals. C; the positive was prepared from the pancreas of EGFP transgenic animals, and D; negative controls were prepared from wild-type virgin pancreatitis-induced animals

**Figure 6:**
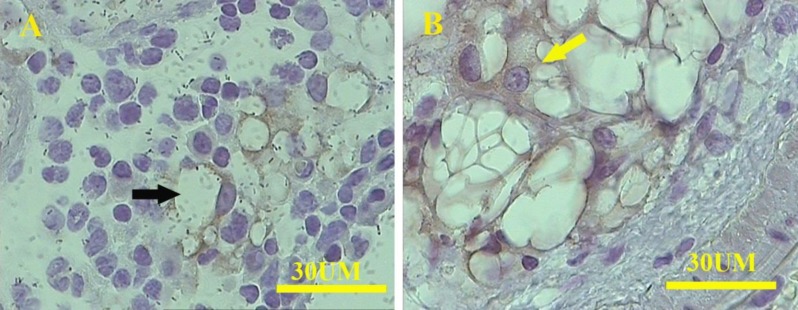
Immunostaining of the pancreas from pregnant pancreatitis-induced animals shows the presence of EGFP-positive cells among the connective-tissue cell populations. They are in the form of adipocytes (black arrow) and mesenchymal cells (yellow arrow)

**Figure 7:**
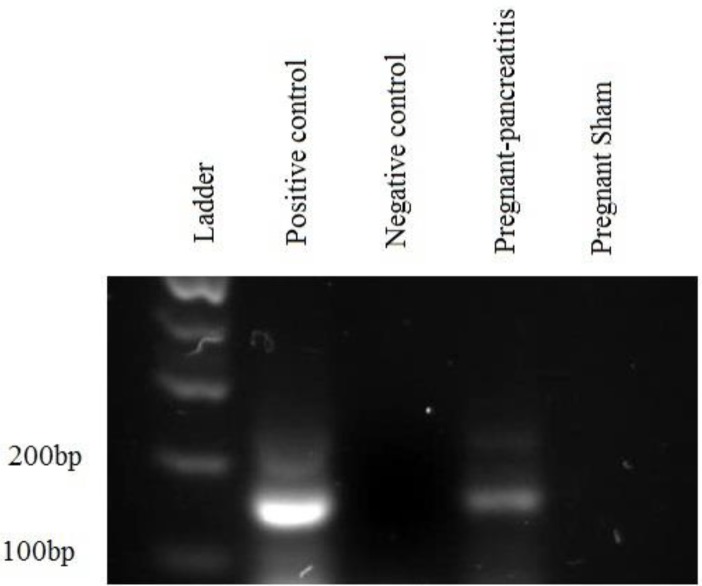
PCR revealed the presence of the EGFP gene in the DNA of cell population extracted from the pancreases of different groups. The pancreata from pregnant pancreatitis-induced animals contain the EGFP gene. Positive control DNA was isolated from pancreata of the EGFP^+^ transgenic animals and negative control was isolated from pancreata of the wildtype animals. The PCR product of EGFP^+^ sample was run in the gel

***Study design***

Sixty mice were divided into three groups. The first group consisted of virgin pancreatitis-induced animals and received caerulein (Sigma, Germany). The second group consisted of pregnant sham animals mated with transgenic GFP mice, and two days after delivery, they received the vehicle (normal saline). The third group consisted of pregnant pancreatitis-induced animals mated with transgenic GFP mice, and two days after delivery, they received caerulein. Each group was divided into two subgroups; the first subgroups were killed 6 hr after the last injection and the second subgroups were kept under the standard conditions for 3 weeks. 

***Induction of acute pancreatitis***

To find the appropriate dose of the caerulein in mice, we designated a pilot study based on the protocol introduced by Lerch and Gorelick, and Warzecha *et al.* in a rodent model (31, 32). To do this, 50, 60, and 80 μg/kg caerulein were injected IP in two mouse strains bulb/c and NMRI. The mice were killed after 6 hr and serological and histological tests were performed. The best dose was chosen to induce pancreatitis in mice.

According to the aforementioned pilot, acute pancreatitis was induced in a mouse model by intraperitoneal injection of 80 μg/kg caerulein, 5 injections at an interval of 1 hr. To confirm the pancreatitis induction, blood samples were taken from the mice 6 hr after the last injection. The mice were killed after 6 hr or 3 weeks; their pancreata were removed, fixed in 10% formalin, sectioned at 5 µm thicknesses, and stained with H&E. The sections were examined under a light microscope (BX41, Olympus, Tokyo, Japan) and a scoring system grade form 0–3 (29) was used to evaluate the severity of pancreatitis.

Leukocyte infiltration (acute and chronic), interstitial edema, vacuolization and gland destruction of pancreatic acinar cells were considered as major criteria in the scoring system. Total white blood cell (WBC) count was evaluated by examining three randomly selected 1 mm^2^ areas of the exocrine pancreas and averaging the scores. The definition of acute and chronic inflammation was also obtained from the same areas based on neutrophil and macrophage/plasma cell counts. The morphological criteria in the scoring system, 0–3, were as follows in all groups: the “0” score means the absence of any histologic finding, score “1” means there is mild inflammation, score “ 2” means there is moderate inflammation, while score” 3” means there is severe inflammation in the pancreas. 

Criteria were defined for scoring levels (0= absent, 1= mild, 2= moderate, 3= severe) for other lexicon components also obtained on three randomly selected 1 mm^2^ areas of the exocrine pancreas and averaging the scores.

***Amylase and lipase activity assay***

The heart blood samples were collected 6 hr after the last injection and centrifuged at 3000 RPM for 10 min; then, the sera were collected for biochemical analysis. Amylase and lipase activity were assessed using an amylase kit (Mancompany, Iran) and a lipase DC kit (Pars Azmun, Iran) according to the manufacturers’ instructions.

***Immunohistochemistry***

Immunohistochemistry analysis of the pancreatic tissue sections was performed using an anti-GFP antibody (Abcam, England). In brief, the pancreas sections at 5 µm thickness were mounted on propylamine-coated slides. The tissue sections were then deparaffinized, and endogenous peroxidase activity was blocked by incubation in 3% H_2_O_2_ in methanol for 10 min(31). For antigen retrieval, the sections were heated in citrate buffer (pH 6.0) (33) at 96 ^°^C for 15 min. Non-specific binding sites were blocked by incubating the sections in PBS containing 10% goat serum for 60 min. The slides were then incubated overnight with primary anti-GFP (Abcam, England) antibody diluted at 1:1000. The slides were gently washed with PBS three times for 5 min and incubated with streptavidin-HRP (Abcam, England) diluted at 1:10000 for 30 min. The slides were washed with PBS twice for 5 min and then incubated with diaminobenzidine chromogen (Sigma, Germany) for 5 min. Finally, the slides were counterstained using hematoxylin for 6 sec, dehydrated in xylene, and mounted.

***Polymerase chain reaction***

To confirm the presence of FMC, the presence of EGFP genes in the mouse mothers, who mated with EGFP transgenic mice, was detected by PCR. DNA was extracted from the pancreas of each animal using a DNA extraction kit (GeNet Bio, Korea) according to the manufacturer’s instructions. DNA concentrations were detected by nanodrop spectrophotometry at 280/260 nm wavelength. Primers were designed by NCBI-Pick primer and primer3 and then analyzed by Allele ID and NCBI-Blast. The sequences of the primers are shown in Table 1. DNA was added to 10X PCR buffer containing 25 mM MgCl_2, _5 unit/µl taq DNA polymerase and 10 mM dNTPs. The initial denaturation time was 10 min at 94 ^°^C with annealing temperatures at 94 ^°^C for 30 sec, 59 ^°^C for 45 sec, and 72 ^°^C for 45 sec. The final extension step was at 72 ^°^C for 10 min. The number of the cycles was 38 times; PCR products were run on agar gel. 

***Statistical analysis***

The mean values of the serum concentrations of amylase and lipase were compared using ANOVA and LSD tests. The data were expressed as mean ±SD. The median value of the pathological score was compared using Kruskal-Wallis and Mann-Whitney tests. The data were expressed as median±range. A *P*-value less than 0.05 was considered as significant difference. The statistical analyses were performed using SPSS 15.0 for Windows (IBM, USA). 

## Results

***Serological findings***

The data from a pilot study in both mouse strains showed that the best dose for pancreatitis induction was 80 µg/kg. However, the balb/C mouse can be considered a superior animal model to the NMRI strain as indicated by the serological tests (Figure 1). The histopathological findings also confirmed the serological data. To prove the acute pancreatitis induction, the mean values of serum levels of amylase and lipase (Figure 2) were analyzed in all groups. The results showed an increase in the serum levels of amylase and lipase in the non-pregnant and pregnant pancreatitis-induced animals compared with pregnant non-induced sham animals (*P*= 0.001).

***Pathological finding***

The histopathological data showed the symptoms of pancreatitis in caerulein-induced animals. These symptoms included leukocyte infiltration into the exocrine part of the pancreas, vacuolization of acinar cells, gland destruction, and edema (Figure 3). The amount of connective tissue showed an increase in pregnant caerulein-induced pancreatitis.

Six hr after the last caerulein injection, the median value of the scoring for acute inflammation was “2”; for chronic inflammation it was “1” and for vacuolization it was “2” for non-pregnant and “1” for pregnant pancreatitis induced animals. In pregnant sham animals, no pancreatitis symptom was observed. 

After 3 weeks, the median value of the scores for acute inflammation was “2” for non-pregnant pancreatitis induced animals. Pregnancy significantly decreased the median value of acute inflammation score to “1” in pregnant pancreatitis animals. Pregnancy led to a significant decrease in the median value of chronic inflammation score from “3” in non-pregnant to “2” in pregnant pancreatitis animals. The scoring value for gland destruction criterion showed a significant reduction from “2” for non-pregnant to “1” in pregnant pancreatitis animals (Figure 4).

***Immunohistochemistry findings***

Immunohistochemistry showed the presence of the EGFP^+^ cells in a limited location of the pancreas in some pregnant animals. The EGFP^+^ cells were involved in the formation of acinar cells in pregnant caerulein-induced pancreatitis (Figure 5). Also, some EGFP^+^ cells were found within the connective tissue near the blood vessels, containing inflammatory leukocytes. The cell morphology indicated the existence of some adipocyte-like cells with one or more lacuna and mesenchymal-like cells with a centrally located nucleus and a few processes in pregnant caerulein-induced animals (Figure 6). In sham animals, a few EGFP^+^ cells were also found within pancreatic acinar cells of some samples. The number of EGFP^+^ cells was much less than those found in pregnant caerulein-induced pancreatitis.

***PCR finding***

PCR assay confirmed the immunohistochemistry and showed the presence of the EGFP gene in the DNA extracted from 20% of the pregnant caerulein-induced animals. In virgin pancreatitis-induced and pregnant sham animals, no band for EGFP was formed. The EGFP-positive animals were used as positive controls (Figure 7). 

## Discussion

This study revealed that pregnancy significantly reduced the score of the induced pancreatitis, and the presence of EGFP-positive cells indicated that the microchimerism transfer from embryo to the mother may result in the regeneration of the exocrine part of the pancreas. Fetal cells have been shown to cross the placental barrier and enter the blood circulation, and then they are implanted in various tissues (17). It has been reported that fetal cell numbers increase in multiparous mothers(20). Pathological changes in the mother’s tissues caused an increase in the number of the fetal cells in the blood and peripheral tissues, and higher numbers of fetal microchimerism have been reported in the chronic state of the injured tissues(17).

Fetal microchimerisms were detected in both normal and injured tissues after pregnancy. The presence of these cells was detected in the normal spinal cord. The cells have been shown to differentiate into neurons as well (20). Human fetal microchimerism has also been shown to differentiate into cardiomyocytes (18). The presence of fetal microchimerism was demonstrated in the pancreas, liver, bone marrow and kidney after diabetes induction. It has been suggested that some multipotent fetal cells migrate into the mouse maternal tissues and differentiate into pancreatic acinar cells, hepatocytes and epithelial tubular cells (27). Besides, ethanol-induced hepatotoxicity and gentamycin-induced nephrotoxicity in mice mothers led to the presence of GFP-positive hepatocytes and epithelial tubular cells originating from the fetuses(17). The results of the current study also confirmed the presence of a few EGFP+ cells in IUI sections of pregnant sham pancreas; however, the number was too limited to detect by PCR. Immunostaining showed that the acinar cells originated from the fetal-derived cells in the pregnant sham and both acinar cells and adipocytes-like cells in the pregnant caerulein-induced animals. The results also showed that the microchimeric cell fate changed by induction of pancreatitis. Increase in FMC number in pregnant caerulein-induced animals may be attributable to FMC proliferation after injury induction. 

The PCR data showed that just 20% of the mice contained the EGFP gene after the first pregnancy. This result was the same as the work performed elsewhere on the spinal cord; however, they found an increase in the frequency of GFP-positive cells in multiparous mice (20). 

There is a controversy about the role of fetal microchimerism in the occurrence of diseases. In diseases such as scleroderma and primary biliary cirrhosis, the presence of fetal microchimerism led to local inflammation reactions (34). In contrast, Johnson *et al.* could not find fetal microchimerism in scleroderma as an autoimmune disease (35), but they reported the presence of such a cell in non-autoimmune diseases (29). Immunostaining results showed the presence of EGFP-positive cells in the connective tissue infiltrated by leukocytes after caerulein induction and pregnancy. These cells were adipocyte-like and mesenchymal-like cells. Such cell could not be identified in the pancreata of the pregnant sham mice. The origin of FMCs is various; trophoblasts, leukocytes and mesenchymal stem cells have been suggested as the origin of microchimerism in the lung (13). Mesenchymal stem cells have been shown to play a critical role in immunomodulation (36) by the production of some cytokines (37). Fetal stem cells have been reported to modify the cytokines in the wound microenvironment of pregnant mice compared with non-pregnant ones (38). The presence of EGFP-positive mesenchymal-like cells may be involved in the reduction in the score of the inflammation process in pregnant pancreatitis-induced compared with virgin-pancreatitis mice and it may be due to the production of immunomodulatory cytokines. 

The beneficial effects of the presence of microchimerism in various diseases were shown previously. Pregnancy has been also shown to prevent systemic sclerosis (39) and plays a role in scarless wound healing (38). The improvement of lupus nephritis was recorded in the patients with fetal microchimerism DNA (40); however, the effects were variable according to the disease stage and sex of the fetus (41). The adverse effects of fetal microchimerism were detected in some other diseases such as hypersensitivity pneumonitis (42). The data of the current study revealed an improvement in the scoring of pancreatitis in the animals experiencing pregnancy compared with virgin ones. 

The limitation of this study was the inaccuracy of the procedure to find a very scant amount of DNA belonging to the embryo in pregnant sham animals. This study also could not find the natural niche of microchimeric cells in the mother’s pancreas. Another limitation of this study was the lack of characterization of the GFP-positive cells with mesenchymal cell phenotype.

## Conclusion

Pregnancy was shown to have a beneficial impact on caerulein-induced pancreatitis. FMCs was shown to differentiate into several cell types including acinar cells in both pregnant sham and caerulein-induced pancreatitis animals and adipocyte and mesenchymal-like cells in pregnant caerulein-induced pancreatitis animals. It seems that the cell populations derived from FMCs depend on the health status of the pancreas. Although pregnancy did not treat pancreatitis completely, it led to improved scoring criterion. 
